# The Neural Basis of Testable and Non-Testable Beliefs

**DOI:** 10.1371/journal.pone.0124596

**Published:** 2015-05-05

**Authors:** Jonathon R. Howlett, Martin P. Paulus

**Affiliations:** 1 Laboratory of Biological Dynamics and Theoretical Medicine, University of California San Diego, La Jolla, California, United States of America; 2 Department of Psychiatry, University of California San Diego, La Jolla, California, United States of America; 3 Laureate Institute for Brain Research, Tulsa, Oklahoma, United States of America; University of New Mexico, UNITED STATES

## Abstract

Beliefs about the state of the world are an important influence on both normal behavior and psychopathology. However, understanding of the neural basis of belief processing remains incomplete, and several aspects of belief processing have only recently been explored. Specifically, different types of beliefs may involve fundamentally different inferential processes and thus recruit distinct brain regions. Additionally, neural processing of truth and falsity may differ from processing of certainty and uncertainty. The purpose of this study was to investigate the neural underpinnings of assessment of testable and non-testable propositions in terms of truth or falsity and the level of certainty in a belief. Functional magnetic resonance imaging (fMRI) was used to study 14 adults while they rated propositions as true or false and also rated the level of certainty in their judgments. Each proposition was classified as testable or non-testable. Testable propositions activated the DLPFC and posterior cingulate cortex, while non-testable statements activated areas including inferior frontal gyrus, superior temporal gyrus, and an anterior region of the superior frontal gyrus. No areas were more active when a proposition was accepted, while the dorsal anterior cingulate was activated when a proposition was rejected. Regardless of whether a proposition was testable or not, certainty that the proposition was true or false activated a common network of regions including the medial prefrontal cortex, caudate, posterior cingulate, and a region of middle temporal gyrus near the temporo-parietal junction. Certainty in the truth or falsity of a non-testable proposition (a strong belief without empirical evidence) activated the insula. The results suggest that different brain regions contribute to the assessment of propositions based on the type of content, while a common network may mediate the influence of beliefs on motivation and behavior based on the level of certainty in the belief.

## Introduction

Belief can be defined as a propositional mental construct that affirms or denies the truth of a state of affairs and is closely linked to basic judgment processes [1]. The maintenance of a large and stable set of beliefs is essential for intelligent behavior, since this forms the basis for any actions which one may take to achieve one’s goals [2]. Beliefs are also frequently used to build mental models of the state of the world and are therefore important constructs to guide decision-making. Dysfunctional belief processing is also likely to play a role in psychiatric illnesses, including psychotic disorders (delusional beliefs) and depression (negative beliefs about self, future and world) [3,4]. Furthermore, patient beliefs about the causes of their illnesses and about potential treatment modalities may also be relevant for treatment [5–7].

Belief states have been modeled using formal mathematical approaches, including Bayesian statistical models. Such models explicitly account for subjective uncertainty about states of the world and have been successfully applied to a number of cognitive domains in humans including multimodal cue integration [8–10], reward learning [11], attentional selection [12], and motor adaptation [13]. The Bayesian approach highlights the complexity of belief processing by taking into account degrees of certainty vs. uncertainty in a proposition, rather than simply considering a binary acceptance or rejection of a proposition. However, the neural processes underlying representations of uncertainty in a [14] proposition are not fully understood. Investigations of neural representations of uncertainty have found that activations differ somewhat depending on the context of the uncertainty. For example, sensory uncertainty is associated with activations in the intraparietal sulcus, anterior cingulate cortex (ACC), and anterior insula [15]. Uncertainty about outcomes (also known as risk when the probabilities are explicitly known) has also been associated with activations in ACC and anterior insula [15]. A meta-analysis found that anterior insula was most consistently associated with risk in fMRI studies [14]. Activity in the medial prefrontal cortex (mPFC) is associated with probability of an expected reward [16].

Beliefs can be categorized by the content of the propositions, and several different categorization schemes are possible. For example, Harris et al. separated beliefs into a number of domains including mathematical, geographical, semantic, factual, autobiographical, ethical, and religious [1]. In a later paper, Harris et al. simply considered religious vs. nonreligious beliefs [17]. Another distinction with a long history in the philosophical literature, but which has received little attention in psychology and neuroscience, is between empirical and non-empirical beliefs. Empirical beliefs are those which could be tested, at least in principle, on the basis of sensory experience [18]. Non-empirical beliefs are held on a different basis, such as intuition or pure logical deduction. Based on this scheme, geographical and factual beliefs would be considered empirical, whereas ethical and religious beliefs would be considered non-empirical. In the remainder of this paper, we refer to empirical beliefs as testable beliefs, and non-empirical beliefs as non-testable beliefs. Because testable and non-testable beliefs are justified by different types of inferences, it is possible that different cognitive and neural systems are involved in these types of beliefs. Notably, however, testable and non-testable beliefs may systematically vary in content; for example, ethical, religious, and metaphysical non-testable beliefs may tend to be more affectively valenced and abstract than objective, factual testable beliefs, which may contribute to processing by differential brain systems. It may also be the case that non-testable beliefs tend to be justified by affective and abstract types of inferences while testable beliefs are justified by affectively neutral and relatively concrete inferences. This suggests that the differences in content and the differences in type of inference may be linked.

Few studies have directly investigated the neural systems underlying the acceptance or rejection of a proposition. In an early neuroimaging study, Harris et al. found that belief, disbelief, and uncertainty regarding a proposition activated distinct neural regions, including prefrontal and parietal cortices and basal ganglia [1]. In particular, belief and disbelief activated ventromedial prefrontal cortex (VMPFC) (for belief) and anterior insula (for disbelief). Uncertainty was associated with increased activation in the dorsal and ventral anterior cingulate cortex (ACC) and decreased activation in the caudate. The authors argued that VMPFC activation may reflect a link between cognition and emotion/reward in the acceptance of a belief, and that insula activation may reflect a negative hedonic reaction to beliefs which are rejected. However, it should be noted that VMPFC is implicated in other functions including a role in the default network and social cognition, which are discussed in more detail below.

In addition to studies focusing directly on belief, there are a number of investigations that are relevant to belief processing. First, there is a growing literature on motivated reasoning, or the tendency of people to accept propositions which result in positive affect while rejecting propositions which result in negative affect (independent of the evidence for or against those assertions). For example, people may disbelieve information that is threatening to a favored political candidate while accepting information that is threatening to an opposing candidate. An imaging study of this phenomenon during a political campaign found that motivated reasoning was associated with activations of VMPFC as well as ACC, posterior cingulate cortex (PCC), insula, and lateral orbital cortex [19]. This study provides evidence that affective influences on belief may be mediated by a specific set of brain areas including VMPFC, ACC and the insula.

Another line of research related to belief has investigated “theory of mind”, a type of social cognition which includes reasoning about the beliefs of others. Such reasoning activates the temporo-parietal junction (TPJ) [20,21]. TPJ appears to be specifically activated when reasoning about the false beliefs of others, rather than true beliefs [22]. Left TPJ was also found to be required to reason about the beliefs of others in a report of brain-damaged individuals [23]. While TPJ appears to be most specifically associated with theory of mind tasks, other regions are often co-activated in these paradigms, including the medial prefrontal cortex (mPFC) [20,21]. A meta-analysis concluded that TPJ is more specifically activated when inferring temporary goals and intentions, while mPFC is more involved in integrating social information over time to develop stable representations of others [24] TPJ and mPFC are also frequently co-activated during the resting state, in the absence of a task [25]. Such resting state activations may represent self-related processing, which occurs in the absence of a task requiring externally directed attention [26]. Furthermore, it has been argued that there is a connection between the self-related processing and theory of mind functions of these regions. Introspective awareness of our own thoughts and beliefs may rely on some of the same cognitive mechanisms as reasoning about the thoughts and beliefs of others [27]. Supporting this idea is the developmental finding that individual children tend to acquire the ability to attribute mental states to self and to others at the same time [28]. In contrast to theory of mind tasks and social cognition, lateral prefrontal cortex is more engaged during non-social cognitive reasoning [29].

As suggested by the studies cited above, it is likely that belief is not a unitary process but is composed of multiple subcomponents. For example, believing or disbelieving a proposition might be processed separately from the level of certainty or uncertainty of a belief. Furthermore, partially separate brain networks may process different types of propositions. As noted above, propositions can be divided between those that can be tested (e.g. “On 9/15/00, the temperature in El Paso, TX was 45°”) and those that cannot (e.g. “Giving love to others is the most important thing in my life”). In the present study, we investigate the neural processes involved in determining whether a proposition is true or false, as well as the level of certainty in that determination, regarding both testable and non-testable propositions. The purpose is to further elucidate the subcomponents of belief processing, which is crucial both in normal behavior and psychiatric conditions.

## Subjects and Methods

### Subjects

The University of California San Diego (UCSD) Institutional Review Board approved this study and all subjects signed informed consent. Fourteen adults gave written consent to participate in this study. Subjects were 20–26 years old (mean 24.4, standard deviation 2.2); 8 were men and 6 were women. All were right-handed English speakers.

### Experimental Design

While in the scanner, subjects were presented with a series of 56 statements. Each trial lasted 12 seconds and began with the presentation of the statement “I believe” with a propositional statement below, which was presented for 1 second, and followed by presenting the available options: “Definitely”, “Possibly”, “Possibly Not”, and “Not at all”. The subject was able to select one of these options once these options were presented on the screen until the end of the trial (12 seconds) by pressing one of four buttons. Once a selection had been made the statements and options were removed and a fixation cross was presented for the remainder of the trial. The list of statements presented was developed for this study [see Appendix]. Statements were divided into two categories: testable statements, which consist of propositions which are either true or false and which can be evaluated by examining existing evidence (e.g. “Hamsters are more common as pets than Turtles”). However, these statements were selected to be relatively idiosyncratic so as to minimize memory and episodic knowledge influences. A second group of statement was denoted as non-testable (e.g. “Giving love to others is the most important thing in my life”). These statements were selected based on ethical, religious, or aphoristic sentiments, which have been used as propositions to guide an individual’s behavior. Each subject was presented with the list of statements in the same order. The list of statements used in this task is included in the appendix.

### fMRI Image Acquisition and Analysis

A fMRI run, which was sensitive to blood oxygenation level-dependent (BOLD) contrast, was collected in a randomized fast-event related design using a Signa EXCITE (GE Healthcare, Milwaukee, Wisconsin) 3.0 Tesla scanner (T2*-weighted echo planar imaging (EPI) scans, TR = 2000 ms, TE = 32 ms, FOV = 230mm³, 64x64 matrix, 30 2.6mm axial slices with 1.4 mm gap, flip angle = 90°, 290 whole-brain acquisitions). fMRI volume acquisitions were time-locked to task onset. A high-resolution T1-weighted image [spoiled gradient recalled (SPGR), TI = 450, TR = 8 ms, TE = 3 ms, flip angle = 12°, FOV = 250mm³, 192x256 matrix, 172 sagittally acquired slices with 1mm thickness] was obtained for anatomical reference.

#### Image analysis

All subject-level structural and functional image processing was done with the Analysis of Functional Neuroimages (AFNI) software package [30]. The multivariate regressor approach detailed below was used to relate changes in EPI intensity to differences in task characteristics [31]. EPI images were co-registered using a 3D-coregistration algorithm [32] that was developed to minimize the amount of image translation and rotation relative to all other images. Six motion parameters were obtained across the time series for each subject. Motion parameters were used as regressors to adjust EPI intensity changes due to motion artifacts. This has been shown to increase power in detecting task-related activation [33]. All slices of the EPI scans were temporally aligned following registration to assure that different relationships with the regressors are not due to the acquisition of different slices at different times during the repetition interval.

Eight individual-specific regressors of interest were generated to delineate the different task conditions (testable vs. non-testable proposition) and response (“Definitely”, “Possibly”, “Possibly Not”, and “Not at all”). To that end, a 0–1 reference function starting from the beginning of the presentation of the propositional statement until the individual selected a response was convolved with a gamma variate function [34] modeling a prototypical hemodynamic response (6–8 second delay [35]) and to account for the temporal dynamics of the hemodynamic response (typically 12–16 seconds) [36]. Jitter was therefore determined by variability in response times across trials. The convolved time series was normalized and used as a regressor of interest. A series of regressors of interest and the motion regressors were entered into the AFNI program 3dDeconvolve to determine the height of each regressor for each subject. The main dependent measure was the voxel-wise normalized relative signal change, or % signal change for short. Images were spatially filtered using a Gaussian Spatial Filter (full-width-half-maximum 4 mm) to account for individual anatomical differences. These data were transformed into Talairach coordinates based on the anatomical MR image for group or second-level analysis. Baseline consisted of the BOLD signal during intertrial intervals and null trials (i.e. fixation on crosshairs without responding).

#### Group level analyses

For fMRI paradigm the dependent measure was the % signal changes during the different task conditions. These dependent measures were entered into a *mixed effects model* [37]. We used the implementation of the linear mixed effects models in R (http://cran.us.r-project.org/), which estimates the parameters of the mixed model using Maximum Likelihood Estimation (MLE). These calculations were done within the R computing environment using routines that read in AFNI data sets. Two LME analyses were performed. In both analyses, condition was modeled as a fixed effect, and subjects were treated as random effects. In the first analysis, acceptance of the proposition was modeled as a fixed effect (with condition as a fixed effect and subjects as a random effect); in the second, certainty was modeled as a fixed effect (with condition as a fixed effect and subjects as a random effect). Analyses were within subject. The effects are estimated using specific contrast matrices. Once these voxel-wise statistics were calculated, we used a threshold adjustment method based on Monte-Carlo simulations to guard against identifying false positive areas of activation. A threshold adjustment method based on Monte-Carlo simulations (AFNI’s program AlphaSim) was applied to guard against identifying false positive areas of activation (considering whole-brain voxel size and 4mm smoothness). For all analyses, AlphaSim identified a minimum cluster volume of 448 μL in conjunction with a cluster significance of p<.005 to result in a voxel-wise probability of p<.01 corrected for multiple comparisons. Average percent signal change was extracted for significant clusters. The fMRI figures were produced using CARET (Computerized Anatomical Reconstruction Toolkit, http://brainvis.wustl.edu/wiki/index.php/Caret:About). In order to assess brain-behavior relationships, the mean difference between response times for testable vs. non-testable propositions was calculated for each subject, which was then tested for correlation with significant clusters for the main effect of condition. The percentage of certain vs. uncertain responses was also calculated for each subject and tested for correlation with significant clusters for the main effect of certainty.

### Behavioral Analysis

Responses were obtained using a four-button response box recorded during each trial to determine response selection (“Definitely”, “Possibly”, “Possibly Not”, and “Not at all”). For the purposes of analysis, each of the four responses was categorized on two dimensions: acceptance and certainty. On the acceptance dimension, “Definitely” and “Possibly” were grouped together as accepting the proposition, and “Not at all” and “Possibly Not” were grouped together as rejecting the proposition. At the same time, for the certainty dimension, “Definitely” and “Not at all” were grouped together as reflecting certainty, while “Possibly” and “Possibly Not” were grouped as reflecting uncertainty. These groupings allowed for separate analyses to investigate acceptance or rejection of a proposition as well as level of certainty in that determination. Response time was also recorded for each trial.

## Results

### Behavioral Data

Different responses occurred with significantly different frequencies, with “Possibly” being the most frequent response (p = 0.02, F = 5.3). “Not at All” was chosen 17% of the time, “Possibly Not” 19%, “Possibly” 44%, and “Definitely” 20%. The interaction between condition and response was not significant.

A main effect of condition on response time was significant, with response times being shorter for non-testable propositions (p < 0.0001, F = 84.2). Mean response time for testable propositions was 6109 msec and for non-testable propositions was 3907 msec. The effect of response type and the interaction between response type and condition were not significant.

### Imaging Data

#### Main Effect of Condition

**Fig 1:**
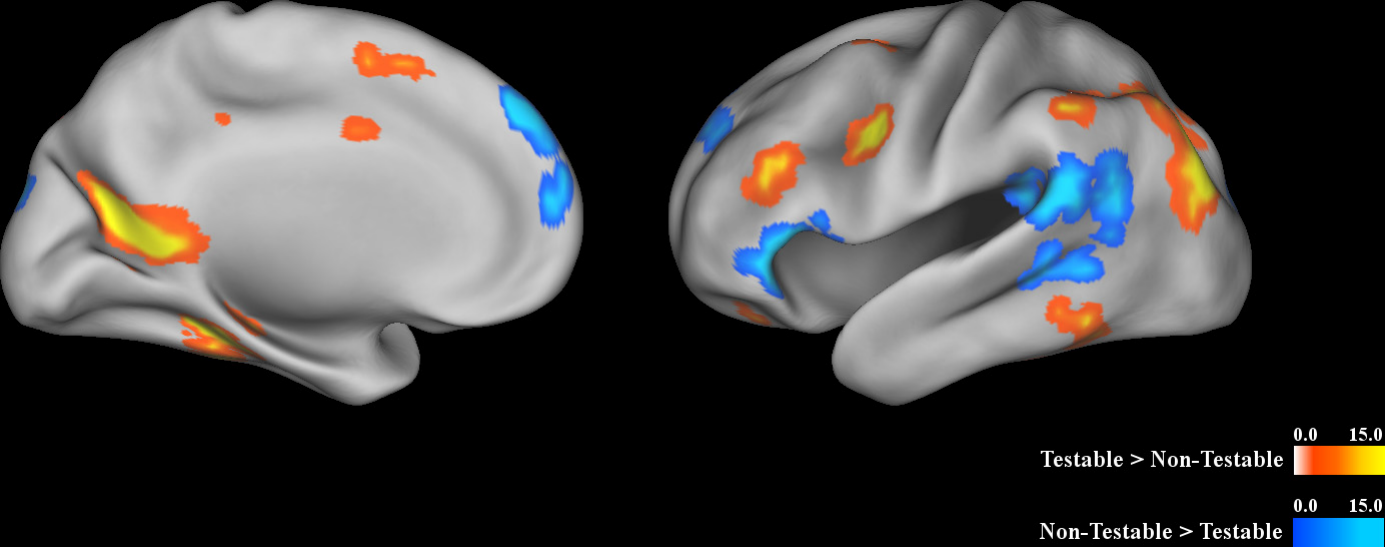
Main effect of condition (testable vs. non-testable). Left medial and left lateral view of clusters which showed a significant effect of condition. Colors represent F-values (blue in regions where activation was greater for non-testable propositions and yellow and orange in regions where activation was greater for testable propositions). Areas activated by testable propositions included dorsolateral prefrontal cortex (DLPFC) and posterior cingulate cortex. Areas activated by non-testable propositions included inferior frontal gyrus, superior temporal gyrus, and an anterior region of the superior frontal gyrus.

Activation was greater for testable than non-testable propositions in bilateral middle frontal gyrus (within dorsolateral prefrontal cortex) (right cluster: x = 43, y = 22, z = 26, Vol. = 10,752 μL, 168 voxels; left cluster: x = -44, y = 30, z = 22, Vol. = 896 μL, 14 voxels) and bilateral posterior cingulate cortex (right cluster: x = 10, y = -54, z = 13, Vol. = 4,352 μL, 68 voxels; left cluster: x = -14, y = -56, z = 12, Vol. = 4,672 μL, 73 voxels). Activation was greater for non-testable than testable propositions in bilateral inferior frontal gyrus (right cluster: x = 48, y = 27, z = 4, Vol. = 512 μL, 8 voxels; left cluster: x = -46, y = 26, z = 0, Vol. = 3,008 μL, 47 voxels), left superior temporal gyrus (x = -53, y = -46, z = 16, Vol. = 8,448 μL, 132 voxels), and bilateral anterior portion of the superior frontal gyrus (right cluster: x = 20, y = 47, z = 35, Vol. = 512 μL, 8 voxels; left cluster: x = -21, y = 49, z = 33, Vol. = 832 μL, 13 voxels). Left lateral and medial views of areas activated by testable and non-testable propositions are shown in Fig 1. Significant clusters for all analyses are shown in Table 1.

**Table 1:** Significant Clusters for All Analyses.

	Cluster Number	Volume (in Voxels)	x	y	z	Region	Brodmann's Area
Main Effect of Condition (Testable vs. Non-Testable)	1	227	37	-62	40	Right Inferior Parietal Lobule	BA 39
	2	168	43	22	26	Right Middle Frontal Gyrus	BA 9
	3	136	-33	-73	34	Left Precuneus	BA 19
	4	132	-53	-46	16	Left Superior Temporal Gyrus	BA 22
	5	73	-14	-56	12	Left Posterior Cingulate	BA 30
	6	68	10	-54	13	Right Posterior Cingulate	BA 23
	7	68	27	1	54	Right Middle Frontal Gyrus	BA 6
	8	59	-31	-36	-15	Left Fusiform Gyrus	BA 20
	9	53	0	16	51	Left Superior Frontal Gyrus	BA 8
	10	47	27	34	-2	Right Middle Frontal Gyrus	BA 47
	11	47	-46	26	0	Left Inferior Frontal Gyrus	BA 47
	12	46	-2	53	36	Left mPFC	BA 9
	13	34	-1	59	13	Left mPFC	BA 10
	14	30	-51	-49	-14	Left Inferior Temporal Gyrus	BA 20
	15	28	-45	2	32	Left Inferior Frontal Gyrus	BA 6
	16	23	-45	-47	41	Left Inferior Parietal Lobule	BA 40
	17	22	-27	1	55	Left Middle Frontal Gyrus	BA 6
	18	14	12	-82	0	Right Lingual Gyrus	BA 18
	19	14	-44	30	22	Left Middle Frontal Gyrus	BA 46
	20	13	19	-75	-29	Right Pyramis	
	21	13	-21	49	33	Left Superior Frontal Gyrus	BA 9
	22	12	2	-41	40	Right Cingulate Gyrus	BA 31
	23	10	-12	-90	23	Left Cuneus	BA 18
	24	9	-25	37	-21	Left Superior Frontal Gyrus	BA 11
	25	9	3	-2	33	Right Cingulate Gyrus	BA 24
	26	8	48	27	4	Right Inferior Frontal Gyrus	BA 45
	27	8	20	47	35	Right Superior Frontal Gyrus	BA 9
	28	7	50	-48	-18	Right Fusiform Gyrus	BA 37
	29	7	8	-54	30	Right Cingulate Gyrus	BA 31
	30	7	5	-71	44	Right Precuneus	BA 7
Main Effect of Acceptance of a Proposition	1	99	13	-72	-6	Right Lingual Gyrus	BA 18
	2	22	-9	-70	25	Left Precuneus	BA 31
	3	20	-14	-47	-17	Left Culmen	
	4	11	17	-83	17	Right Cuneus	BA 18
	5	9	29	-85	13	Right Middle Occipital Gyrus	BA 19
	6	9	-20	46	15	Left Superior Frontal Gyrus	BA 10
	7	7	-1	23	17	Left Anterior Cingulate	BA 24
Condition by Acceptance Interaction	1	18	-48	26	8	Left Inferior Frontal Gyrus	BA 45
Main Effect of Certainty	1	123	-1	6	7	Left Caudate	
	2	46	-45	-67	19	Left Middle Temporal Gyrus	BA 39
	3	42	-2	45	29	Left mPFC	BA 9
	4	26	-3	-49	8	Left Posterior Cingulate	BA 29
	5	24	-1	52	8	Left mPFC	BA 10
	6	24	46	-67	14	Right Middle Temporal Gyrus	BA 39
	7	24	-2	-1	42	Left Cingulate Gyrus	BA 24
	8	23	-5	-48	46	Left Precuneus	BA 7
	9	20	46	-21	44	Right Postcentral Gyrus	BA 2
	10	19	17	-83	17	Right Cuneus	BA 18
	11	17	-12	-70	-3	Left Lingual Gyrus	BA 18
	12	12	4	-65	5	Right Lingual Gyrus	
	13	11	-7	-51	30	Left Precuneus	BA 31
	14	10	-3	-22	46	Left Paracentral Lobule	BA 31
	15	7	-36	-15	-3	Left Claustrum	BA 13
Condition by Certainty Interaction	1	31	-51	4	-13	Left Superior Temporal Gyrus	BA 21
	2	24	-40	-10	-1	Left Insula	BA 13
	3	22	-62	-40	-1	Left Middle Temporal Gyrus	BA 21
	4	18	-30	-7	8	Left Lentiform Nucleus	BA 13
	5	12	-25	-50	-14	Left Culmen	BA 37
	6	11	-24	-92	-7	Left Inferior Occipital Gyrus	BA 18
	7	11	15	52	31	Right Superior Frontal Gyrus	BA 9
	8	10	42	-21	5	Right Insula	BA 13
	9	9	27	-10	3	Right Lentiform Nucleus	
	10	9	-51	-26	11	Left Transverse Temporal Gyrus	BA 41
	11	9	-47	-58	12	Left Middle Temporal Gyrus	BA 39
	12	8	-44	-23	-19	Left Fusiform Gyrus	BA 20
	13	8	-41	22	-12	Left Inferior Frontal Gyrus	BA 47
	14	8	37	-74	-7	Right Middle Occipital Gyrus	BA 19
	15	8	62	-30	12	Right Superior Temporal Gyrus	BA 42
	16	8	-43	-54	27	Left Supramarginal Gyrus	BA 39
	17	7	-25	-20	-23	Left Parahippocampal Gyrus	BA 36
	18	7	44	9	-1	Right Insula	BA 13
	19	7	50	-35	18	Right Insula	BA 13
	20	7	2	-79	21	Right Cuneus	BA 18

#### Main Effect of Acceptance of a Proposition

**Fig 2:**
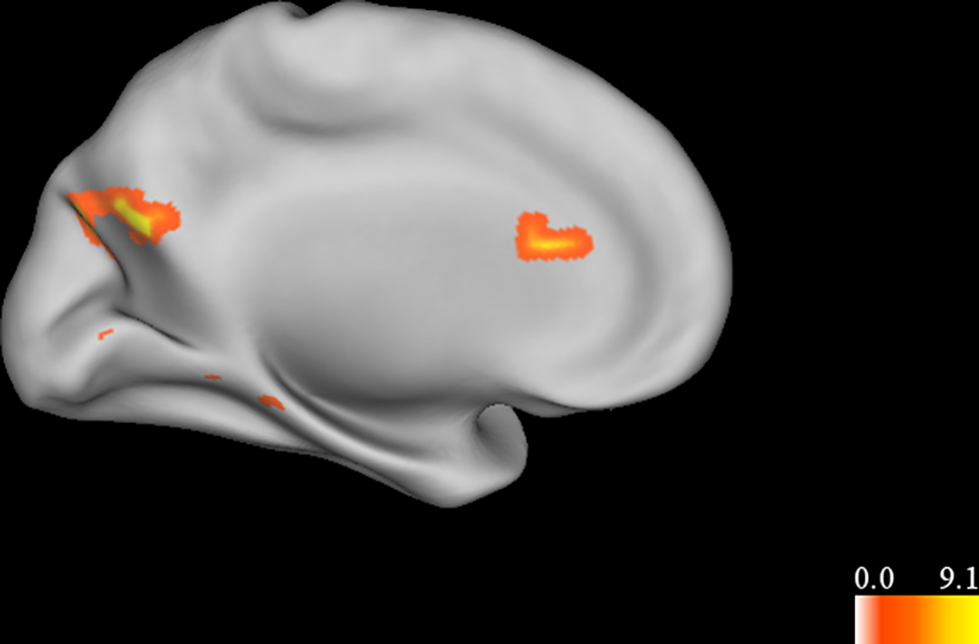
Main effect of rejection of a proposition. Left medial view of clusters which were significantly activated when a proposition was rejected. Colors represent F-values. Areas which were more active when rejecting a proposition include the dorsal anterior cingulate cortex (ACC). No areas were significantly more active when accepting a proposition.

No areas were more active when accepting than when rejecting a proposition. Areas that were more active when rejecting than accepting a proposition included left dorsal ACC (x = -1, y = 23, z = 17, Vol. = 448 μL, 7 voxels) and left precuneus (x = -9, y = -70, z = 25, Vol. = 1,408 μL, 22 voxels; Fig 2).

#### Condition by Acceptance Interaction

**Fig 3:**
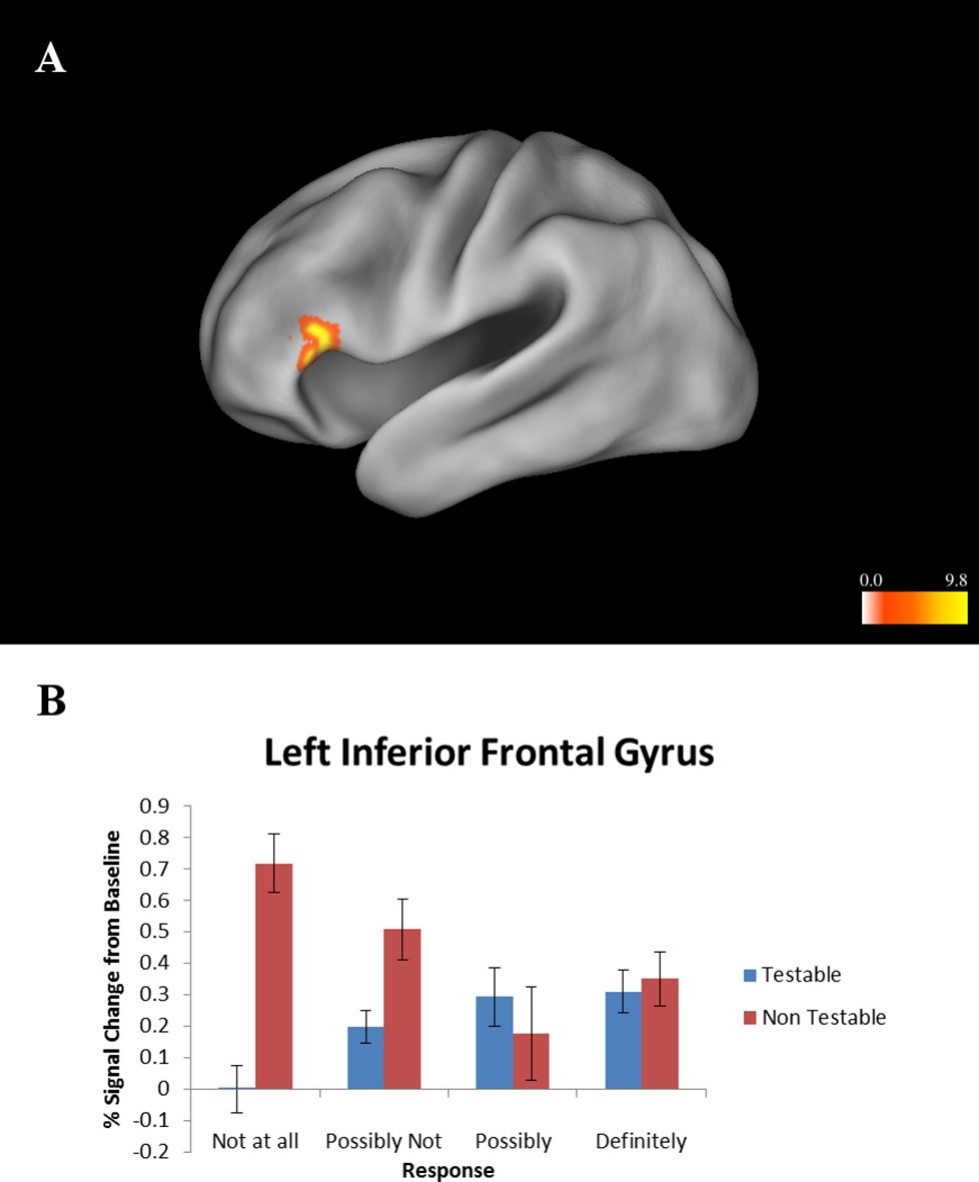
Interaction between acceptance of a proposition and condition (testable vs. non-testable). (A) Left lateral view. Colors represent F-values. (B) Activation levels of inferior frontal gyrus cluster shown in Fig 3A. This region was most active when rejecting a non-testable proposition. Error bars represent standard error of the mean.

LME indicated a significant condition by acceptance interaction in left inferior frontal gyrus (x = -48, y = 26, z = 8, Vol. = 1,152 μL, 18 voxels). Specifically, this region was more active when a non-testable proposition was rejected, but less active when a testable proposition was rejected (Fig 3).

#### Main Effect of Certainty

**Fig 4:**
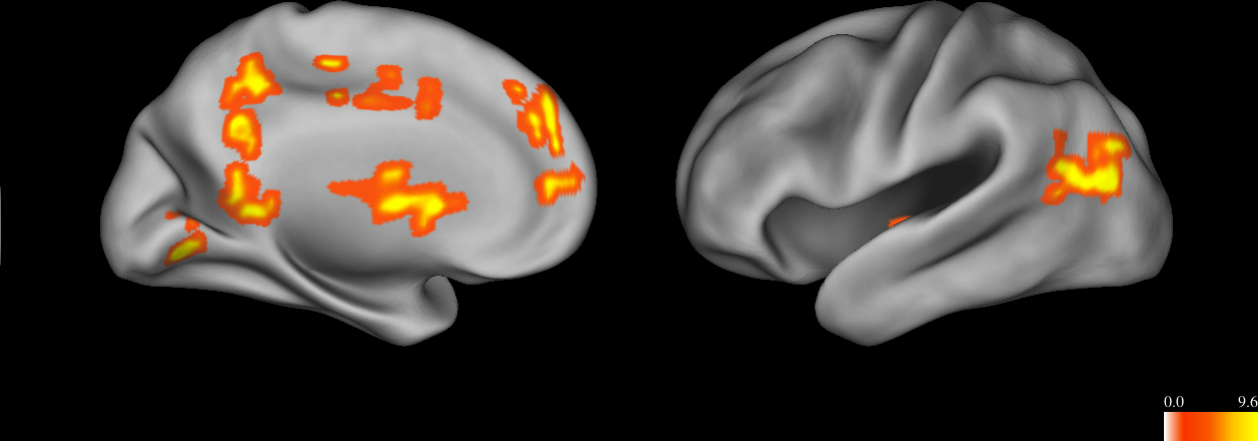
Main effect of certainty. Colors represent F-values. Regions which were more active when a proposition was deemed certainly true or false (“Definitely” or “Not at all”) included medial prefrontal cortex, caudate, posterior cingulate gyrus, and a region of the temporal lobe near the temporo-parietal junction. No areas were more active during uncertainty.

When subjects were certain that a proposition was either true or false, activation was greater in a region of the left mPFC (x = -2, y = 45, z = 29, Vol. = 2,688 μL, 42 voxels), left caudate (x = -1, y = 6, z = 7, Vol. = 7,872 μL, 123 voxels), left posterior cingulate (x = -3, y = -49, z = 8, Vol. = 1,664 μL, 26 voxels), and bilateral middle temporal gyrus (right cluster: x = 46, y = —67, z = 14, Vol. = 1,536 μL, 24 voxels; left cluster: x = -45, y = -67, z = 19, Vol. = 2,944 μL, 46 voxels; Fig 4). There were no regions that were more active when subjects were uncertain that a proposition was true or false.

#### Condition by Certainty Interaction

**Fig 5:**
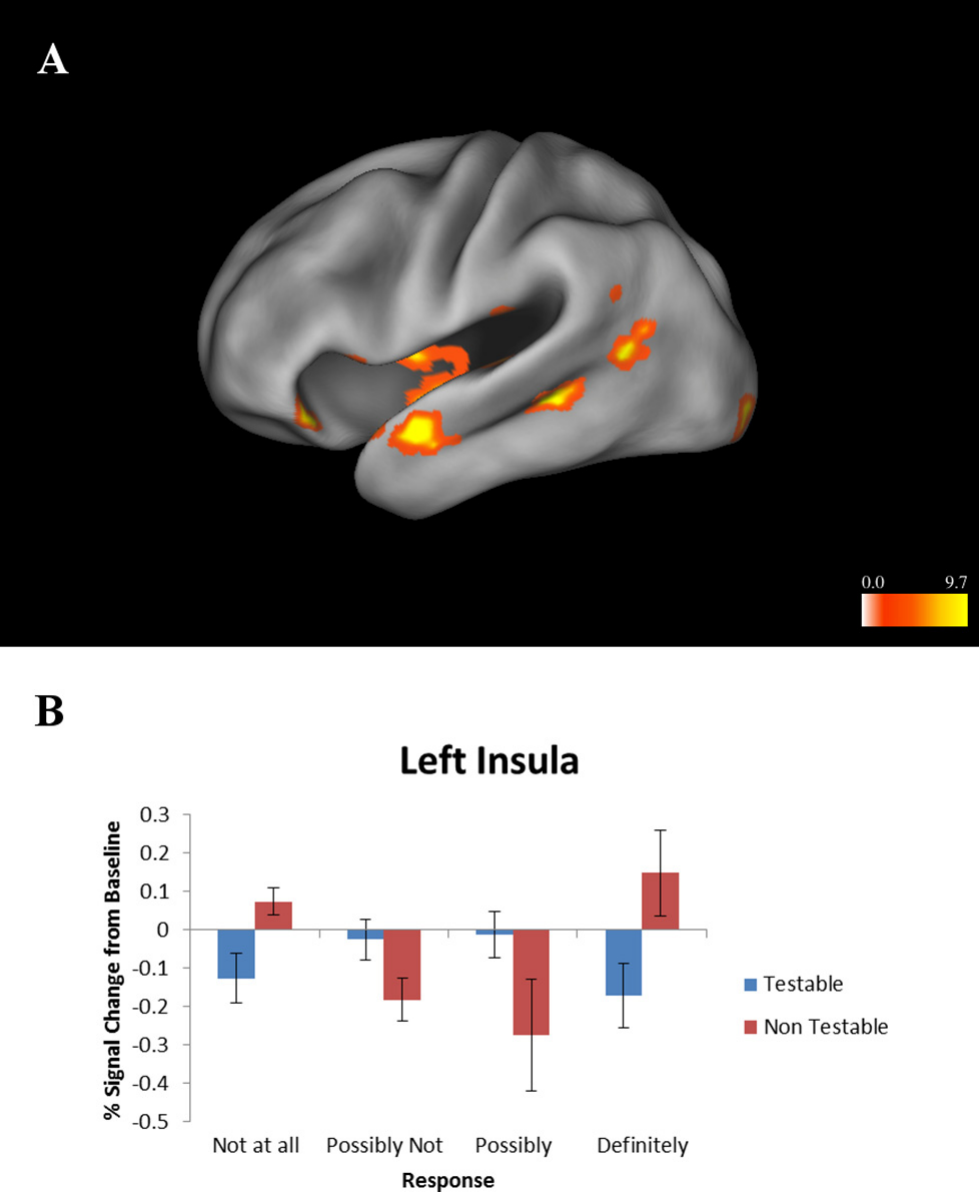
Interaction between certainty and condition (testable vs. non-testable). (A) Left lateral view. Colors represent F-values. All areas were more active during certainty that a non-testable proposition was true or false. (B) Activation levels of insula cluster as shown in Fig 5A. This region was more active when a non-testable proposition was deemed certainly true or certainly false. Error bars represent standard error of the mean.

There was a significant condition by certainty interaction in bilateral insula (right cluster: x = 44, y = 9, z = -1, Vol. = 448 μL, 7 voxels; left cluster: x = -40, y = -10, z = -1, Vol. = 1,536 μL, 24 voxels). The insula was more active during certainty that a non-testable proposition was true or false, but less active during certainty that a testable proposition was true or false (Fig 5). No relationships were found between brain activation and individual differences in responses or reaction times. There was no correlation between mean difference in response time for testable vs. non-testable propositions for each subject and activity within significant clusters from the main effect of condition, and no correlation between percent certain vs. uncertain responses and activity within significant clusters from the main effect of certainty.

## Discussion

This study investigated the neural processes involved in evaluating the truth or falsity of different types of propositions, as well as the level of certainty in that determination. Propositions were divided into testable and non-testable statements, consistent with the idea that belief processing may differ depending on the type of statement being evaluated, rather than being a unitary process. Results indicated that processing testable statements activated areas including DLPFC and posterior cingulate cortex, while processing non-testable statements activated areas including inferior frontal gyrus, superior temporal gyrus, and an anterior region of the superior frontal gyrus. While no areas were more active when accepting a proposition, the anterior cingulate cortex was activated when rejecting a proposition (regardless of whether the proposition was testable or not). The inferior frontal gyrus was specifically activated when rejecting a non-testable proposition. A number of areas were more active when the subject was certain that a proposition was either true or false, including medial prefrontal cortex, the caudate, the posterior cingulate, and a region of middle temporal gyrus near the temporo-parietal junction. The insula was more active when the subject was certain that a non-testable proposition was true or false.

Areas activated selectively by testable propositions included DLPFC and posterior cingulate. This may reflect the function of DLPFC in working memory [38] and the manipulation of information [39], which may have been required to assess the geographical, demographic, and other types of testable statements based on subjects’ preexisting knowledge. Similarly, the posterior cingulate cortex is known to be involved in memory retrieval [40], which may also be required when processing these types of statements. The greater engagement of memory resources and manipulation of information for testable statements is also consistent with the behavioral finding that response times were longer for testable statements. Contrastingly, non-testable propositions activated inferior frontal gyrus, superior temporal gyrus, and a very anterior region of the prefrontal cortex (dorsomedial prefrontal cortex). Both inferior frontal gyrus and superior temporal gyrus have been implicated in semantic processing [41–43]. This type of processing can also be understood as conceptualization based on prior experiences, which is one function of the default network [44]. Accounts of prefrontal cortex function have suggested that the most anterior part of the prefrontal cortex is specialized for the most abstract level of reasoning, planning, and problem-solving [45,46]. One somewhat speculative explanation of these findings is that a combination of semantic processing/conceptualization and abstract reasoning may be necessary for judging non-testable propositions. It should also be noted that the region of anterior prefrontal cortex referenced above is near a region of mPFC which, in combination with the superior temporal gyrus, has been implicated in social mentalizing and belief reasoning about other persons [21].

The rejection of a proposition was associated with activity in the dorsal anterior cingulate cortex (ACC). Dorsal ACC has been implicated in monitoring cognitive conflict, error signaling, and the engagement of cognitive control [47,48]. It is also more active during cognitive conflict in social cognition [49]. In this context, the dorsal ACC may be responding to the conflict or incompatibility between a proposition and the subjects’ pre-existing knowledge and beliefs, which ultimately lead to the proposition being rejected. Previous accounts of belief processing have suggested that rejecting a proposition may require greater cognitive control than accepting it [1]. The interaction between acceptance and condition showed that inferior frontal gyrus was specifically activated when rejecting a non-testable proposition. As noted above, this region has been associated with semantic processing [41]. It has also shown a role in inhibitory control [50]. The region’s activity in this context therefore suggests that it may be suppressing a default response to accept a non-testable proposition, consistent with the idea that rejecting a proposition requires more cognitive control than accepting one. Together, these results suggest that deciding to accept or reject a proposition may involve a determination of whether the proposition conflicts with pre-existing knowledge and beliefs. In the absence of any conflict, the default response is to accept the proposition. If the proposition conflicts with pre-existing beliefs, cognitive control processes are activated to override the default response, leading to the proposition being rejected. The rejection of a proposition therefore requires more processing resources than the acceptance of a proposition.

While no regions were more active during uncertainty (“Possibly” or “Possibly Not”), a number of areas were activated when subjects were certain that a proposition was true or false. It may be seen as surprising that certainty engages more brain regions than uncertainty, given that it might be expected that more resources are needed when it is difficult to determine whether a proposition is true or false. In this case, the increased activity seen during certainty may represent the mobilization of motivational areas in response to a proposition being deemed certainly true or false. Areas which were more active during certainty included medial prefrontal cortex and the caudate, which are known to be involved in the evaluation of stimuli and in reward, suggesting that certainty in a belief may represent an evaluative judgment which activates brain areas involved in other types of evaluations [46,51]. These regions were activated in combination with the posterior cingulate gyrus and a region of the temporal lobe near the temporo-parietal junction. This group of regions is similar to a network which has been identified as playing a role in mentalizing, which includes reasoning about the beliefs of others, as well as during “resting” conditions when subjects process self-relevant information [52].

Certainty that a non-testable belief was true or false was associated with activation of the insula. This region has been associated with interoception, or processing of internal cues about bodily state which then influence subjective feelings [53]. This subjective feeling state may be involved in feeling certain in a belief without evidence, which could be described as “faith” or a “gut feeling”. Testable beliefs may rely more on DLPFC processing rather than interoception and therefore may not engage the insula. We have recently proposed a refinement of the interoceptive model of anxiety, which incorporates belief-related processing [54]. In this model, beliefs contribute to the evaluation of the anticipatory interoceptive signals, which become exaggerated in valence and are evaluated relative to the individual’s internal state. As a consequence, sub-threshold afferent interoceptive signals are amplified and associated with the prediction of potential aversive or negative beliefs. Moreover, when anxiety prone individuals receive body signals they cannot easily differentiate between those that are associated with potential aversive (or pleasant) consequences versus those that are part of constantly ongoing and fluctuating interceptive afferents. Thus, these individuals imbue afferent interoceptive stimuli with motivational significance, i.e. an increased tendency to plan and act upon the reception of this input. In the context of belief processing, non-testable beliefs may be relatively independent of DLPFC-related reasoning process and may have important influences on behavior.

Taken together, these findings suggest that the systems evaluating testable and non-testable propositions are partially separable, while still recruiting common neural circuitry. Specific brain regions may be separately recruited based on the distinct demands of processing testable propositions (requiring working memory and the manipulation of information) and non-testable propositions (semantic processing/conceptualization and abstract reasoning). However, both types of statements may ultimately be evaluated by a set of evaluative brain areas, which overlap with the regions which process the beliefs of other people. These regions may mediate the influence of beliefs on subsequent behavior by connecting them with motivational systems.

Our findings include some similarities and differences with those of Harris et al., who used a similar yet distinct experimental design [1]. In particular, the previous study found VMPFC activity when a proposition was accepted, while the present study found no areas, which were more active when a proposition was accepted. However, there was similarity in that the previous study found decreased caudate activity during uncertainty, while the present study found caudate activation during certainty. The differences in findings may be related to the different study design; in the previous study the possible responses for the subject were “true”, “false”, and “uncertain”, while in the present study the responses were “Definitely”, “Possibly”, “Possibly Not”, and “Definitely Not”. This could have caused the subjects to process the propositions differently. It is also important to note that the previous study used propositions from a mixture of domains including mathematical, geographical, semantic, factual, autobiographical, ethical, and religious, and that a distinction was not made between testable and non-testable beliefs. Further research is needed to elucidate the processing of belief propositions in different contexts.

There are some limitations to the current study, which must be considered when interpreting the results. First, because a larger number of propositions were accepted than rejected (64% were accepted), it is possible that a subsequent decrease in statistical power for rejection events resulted in false negative results. Second, as acknowledged above, it is by no means the case that testable vs. non-testable is the only way to categorize the content of propositions. However, it is likely that the type of evidence required to evaluate a proposition (empirical vs. non-empirical) will influence the neural processing of the proposition, by determining which cognitive processes are engaged in evaluating this evidence. Testability may therefore be an important dimension to consider when developing a richer understanding of belief.

Another limitation is that subjects may have interpreted the propositions and the responses differently. For example, some subjects may differ in their interpretation of which propositions are testable and which are not. Furthermore, subjects may differ in their understanding of the precise meanings of the responses (e.g. “Definitely” vs. “Possibly”). Future research could explore these differences by asking subjects to rate the propositions as testable or non-testable. Responses could also be specified in terms of probabilities to represent uncertainty, rather than verbal terms. Additionally, subjects could be asked to rate propositions on affective dimensions and abstractness, to explore the connection between these dimensions and neural activity.

While belief is clearly an important cognitive process with important implications for behavior and psychopathology, many aspects of belief processing remain poorly understood. The present study focuses on brain systems underlying processing of different types of belief inference as well as levels of certainty in beliefs. It supplements previous research, which suggests that different types of beliefs may recruit specialized brain regions, while at the same time a common set of evaluative brain areas mediates the influence of beliefs on behavior through the brain’s motivational systems. Some of these areas belong to the default network which is more generally involved in mentalizing and social cognition. A fuller understanding of these processes may not only shed light on normal functioning, but also abnormal belief processing in psychopathology.

## Appendix

**Table:** Testable Propositions

Honda has greater revenue than AT&T
Chevron has greater revenue than Samsung
Karen is a more common first name than Nancy
Philadelphia has more inhabitants than Phoenix
Charles is a more common first name than Richard
Bolivia has a greater GDP than Ethiopia
Women carpool more often than men
Birmingham AL is east of Indianapolis IN
Roseate Spoonbill is larger than Red-tailed Hawk
In 1810 Boston was larger than Baltimore
Hamsters are more common as pets than Turtles
More people commute between 630-700a than 730-800a
On 9/15/00 the temperature in El Paso TX was 45
In 1950 Los Angeles was larger than Philadelphia
Hookworms are found in at least 500 million people
More men work in arts, entertainment and recreation
Dolphins and humans share a common ancestor
Relatively more whites are in Nebraska than Colorado
Nashville TN has more inhabitants than Raleigh NC
The roots of a rye plant can spread up to 400 miles
American Paddlefish are larger than Blue Catfish
More HS graduates live in New Mexico than Tennessee
Missouri has relatively more older adults than Wisconsin
Thailand has a greater GDP than Venezuela
Wichita KS is east of Oklahoma City OK
Columbus OH is north of Philadelphia PA
On 4/12/97 the temperature in Fairbanks AK was 39
There are more female foreign born naturalized citizens

**Table:** Non-Testable Propositions

Life has no meaning
The human spirit is immortal
Life is like a journey
There is a God or gods
There is life after death
Life is planned out for you
Most events in my life are due to chance
There is nothing beyond my material existence
I have a spirit or soul that can survive my death
I believe there is a personal God
The most important thing in life is to be good
There are forces of evil in the Universe
There are forces in the universe
This world is full of suffering
I believe in reincarnation
God is an all pervading presence
I believe that there is heaven
God is a life force
What happens after I die is determined by how I lived my life
Giving love to others is the most important thing in my life
I believe that there is a purpose in life
Science will eventually explain everything
It is important to receive love
Physical well being is linked to spiritual well being
It is possible that other worlds exist
Human physical contact can be a spiritual experience
Everything happens for a reason
Everything has come about through evolution

## Supporting Information

S1 Dataset Acceptance by condition clusters. List of significant clusters for the acceptance by condition interaction.
(CSV)

S2 Dataset Acceptance by condition activity. Mean % signal change and standard error of the mean for each significant cluster for the acceptance by condition interaction.
(CSV)

S3 Dataset Acceptance main effect clusters. List of significant clusters for the main effect of acceptance.
(CSV)

S4 Dataset Acceptance main effect activity. Mean % signal change and standard error of the mean for each significant cluster for the main effect of acceptance.
(CSV)

S5 Dataset Certainty by condition clusters. List of significant clusters for the certainty by condition interaction.
(CSV)

S6 Dataset Certainty by condition activity. Mean % signal change and standard error of the mean for each significant cluster for the certainty by condition interaction.
(CSV)

S7 Dataset Certainty main effect clusters. List of significant clusters for main effect of certainty.
(CSV)

S8 Dataset Certainty main effect activity. Mean % signal change and standard error of the mean for each significant cluster for the main effect of certainty.
(CSV)

S9 Dataset Condition main effect clusters. List of significant clusters for main effect of condition.
(CSV)

S10 Dataset Condition main effect activity. Mean % signal change and standard error of the mean for each significant cluster for the main effect of condition.
(CSV)
